# Anticorrosion Superhydrophobic Surfaces on AA6082 Aluminum Alloy by HF/HCl Texturing and Self-Assembling of Silane Monolayer

**DOI:** 10.3390/ma15238549

**Published:** 2022-11-30

**Authors:** Amani Khaskhoussi, Luigi Calabrese, Edoardo Proverbio

**Affiliations:** Department of Engineering, University of Messina, Contrada di Dio Sant’Agata, 98166 Messina, Italy

**Keywords:** super-hydrophobicity, self-assembling, aluminum alloy, surface treatment, corrosion

## Abstract

In this paper, the tailoring of superhydrophobic surfaces on AA6082 aluminum alloy by chemical etching in an HF/HCl solution, followed by silane self-assembling, was applied for enhanced corrosion protection in the marine field. In particular, different etching times were considered in order to optimize the treatment effect. The results indicate that all the prepared surfaces, after silanization, were characterized by superhydrophobic behavior with a contact angle higher than 150°. The contact and sliding angles strongly depend on the surface morphology at varying etching times. The optimum was observed with an etching time of 20 s, where a microscale coral-like structure coupled with a homogeneous and ordered pixel-like nanostructure was obtained on the aluminum surface showing a Cassie–Baxter superhydrophobic behavior with a water contact angle of 180° and a sliding angle equal to 0°. All superhydrophobic surfaces achieved an enhanced corrosion protection efficiency and impedance modulus up to two orders of magnitude higher than the as-received AA6082 in simulated seawater.

## 1. Introduction

Aluminum and its alloys are attractive candidates as engineering materials for civil applications, the electronics industry, automotive, marine and aerospace engineering for their excellent performance characteristics, such as excellent formability, high strength-to-weight ratio, good machinability and relatively low cost [1,2]. In particular, the use of aluminum alloys as weightless components in the marine industry has a massive effect not only in production expenses but also in life-cycle costs [3,4]. However, a relevant problem is the high sensitivity of aluminum alloys to corrosion in seawater [5,6]. In order to overcome this issue, a significant effort has been made in the research field of anti-corrosion coatings such as sol-gel coating [7,8], chemical vapor deposition coating [9,10] and electrochemical deposition coating [11].

Surface modification and superhydrophobic coatings represent a promising research frontier to obtain corrosion-resistant tailored functional aluminum surfaces, with specific behavior such as anti-icing [12], anti-fouling [13], self-cleaning [14] and anti-bioadhesion [15,16].

Surface super-hydrophobicity is based on the low interaction of water with the solid leading to a water contact angle (WCA) higher than 150° and a water sliding angle (WSA) less than 5° [15,17,18,19,20,21,22] A two-step surface modification approach has been proposed in the literature: (i) Fabrication of a hierarchical micro/nanostructure and (ii) Surface functionalization in order to reduce the surface energy [23]. In recent years, some works have addressed the fabrication of superhydrophobic surfaces on different aluminum alloys [14,24,25,26]. To meet demand for the industrial application of such alloys, an environmentally acceptable process with an easy, economic and time-saving procedure is still required. In this context, chemical etching to create a rough textured surface followed by coating with a low-surface energy material, such as alkyl and perfluoroalkyl silanes [27] and fatty acids [28], is one of the most economical and environmentally suitable methods that permits a high level of flexibility and ease of application [29,30]. Wet chemical etching of aluminum alloys has been reported in numerous papers [14,31,32,33], by immersion in metal chloride solutions (e.g., FeCl_3_ [34], NaCl [35], CuCl_2_ [36]) or in alkaline solutions (e.g., NaOH [37]). Acids, such as H_2_SO_4_ and HCl, or aqueous acidic solutions, such as HCl/H_2_O_2_ [38] or HCl/HF (Beck’s solution) [39,40], were also used for aluminum etching. Sarkar et al. [41] elaborated a superhydrophobic surface on aluminum substrate by an etching step followed by film deposition; however, the control of the preparation conditions was extremely critical. Analogously, Saleema et al. [42] prepared a superhydrophobic aluminum surface with a water contact angle (WCA) of about 162° and a water sliding angle (WSA) of about 4°, evidencing that due to the high cost, the process cannot be easily applied in industrial use. Zhang et al. [43] fabricated a superhydrophobic surface by immersion of an aluminum alloy sample into a solution of hydrochloric acid (350 g/L) and lauric acid (20 g/L) at a temperature of 50°C for 10 min; the heating system used was quite complex. Weibel et al. [44] obtained a superhydrophobic surface on an aluminum substrate (WCA = 165°/WSA < 3°) by a multistep procedure: etching with HCl solution followed by treatment with trimethoxy(propyl)silane and coating with PTFE by a physical vapor deposition method. In order to overcome the applicability limitations observed in the above reported procedures, a simple process consisting of the etching of an aluminum substrate by Beck’s solution and subsequent surface modification with fluoroalkyl silane was proposed by Qian and Shen [39]. The optimization of the preparation conditions, such as the etching time and its relationship with surface morphology and superhydrophilicity, represent a key research point in this topic. Furthermore, a relevant aspect that needs to be accurately taken into account is to achieve a surface with the required barrier and corrosion protection in such severe environmental conditions as seawater to improve its applicability in the marine industrial field. Consequently, an increase in knowledge is required to understand and predict the behavior of superhydrophobic surfaces in such an environment, coupling morphological, physical and barrier surface properties.

In this concern, Beck’s solution, originally used in metallography for the dislocation and revealing of grain boundaries, could represent a promising etchant to obtain superhydrophobic textured surfaces which potentially would be effective for enhanced corrosion protection [45,46,47]. In the present study, the Beck’s solution was used to modulate the surface texture on an AA6082-T6 aluminum alloy substrate. The etched aluminum surfaces were coated with a fluoro-free self-assembled silane film to achieve super-hydrophobicity. The surface morphology was investigated in-depth to explore the correlation between the etching time and the surface wetting behavior. Furthermore, potentiodynamic and electrochemical impedance spectroscopy (EIS) measurements were performed with increasing etching times to assess the anti-corrosion performance of the superhydrophobic layers.

## 2. Materials and Methods

### 2.1. Materials

Aluminum plates of EN AW-6082 T6 alloy were used as substrates. The composition of this aluminum alloy, obtained by EDS analysis, is detailed in Table 1.

Hydrochloric acid (HCl, 37% concentration) was purchased from Honeywell Fluka (Austria). Hydrofluoric acid (HF, 48% concentration) and Octadecyltrimethoxysilane (C_21_H_46_O_3_Si, 90%) were acquired from Sigma-Aldrich (St. Louis, MO, USA),while Toluene was obtained from Riedel-de Haën (Seelze, Germany). Ethanol (99%) and acetone were purchased from J.T. Baker (Phillipsburg, NJ, USA). Ultra-pure water (conductivity ≤ 0.1 μS/cm) from Best Chemical (Italy) was used throughout the experiments.

### 2.2. Fabrication of Superhydrophobic Surfaces

Strips with dimensions of 30 × 24 × 2 mm^3^ were obtained from the as-received plate. No grinding or lapping of the as-received surface was performed. The aluminum substrates were then rinsed ultrasonically with ethanol, acetone and ultra-pure water and dried at room temperature. A two-step process was performed to create the superhydrophobic surfaces: (i) Etching step. Initially, roughness was created on the aluminum samples by chemical etching in a Beck’s acid solution (73 vol.% HCl, 5 vol.% HF and 22 vol.% bi-distilled water) for the following times: 5 s; 10 s; 15 s; 20 s and 30 s. Then, the etched samples were rinsed with bi-distilled water and cleaned in an ultrasonic bath with ultra-pure water to remove residual acids and dried at 70° for 60 min; (ii) Silanization step. The resulting aluminum substrates were immersed in a 0.1 wt.% solution of Octadecyltrimethoxysilane (S18) in a toluene solvent for 10 min in order to obtain a thin film deposition (nanometric thickness). Afterward, substrates were vertically dried for 3 h at 100 °C to complete the silane curing.

Table 2 summarizes the sample preparation details and codes for the different preparation conditions. The etched surfaces are coded with the prefix “A_F”. The S letter is added if the silanization (step 2) is performed. Finally, a number indicating the etching time is applied. For example, the A_FS5 code refers to the aluminum sample etched for 5 s and then coated with a self-assembled silane layer. Furthermore, as a reference, the as-received surfaces (silanized and not, coded as A_RS and A_R, respectively) are considered. 

### 2.3. Sample Characterization

Wettability measurements were performed using a tensiometer instrument (Attension, Biolin Scientific, Gothenburg, Sweden) according to the sessile drop method. A 3 μL ultrapure water droplet was automatically set on the surface of the sample at room temperature. The droplet image was recorded by a micro CCD camera and evaluated by shape analyzer PC Attension software. The measurements were completed for different surface areas on each sample. The sliding angle measurement was performed to determine the critical angle when a 3 μL liquid droplet begins to slide down an inclined plate by using a digital goniometer set-up. Six replicas were performed for each condition.

Scanning Electron Microscopy (SEM-FIB Zeiss Cross Beam 540, ZEISS, Obwerkochen, Germany) was used to check the surface morphologies of the different sample surfaces. Three images at different magnification were used to calculate the geometrical parameters for each sample. These scanning electron microscope images were analyzed based on the combination of two software applications—GIMP and ImageJ: GIMP was used to adjust the contrast of the images to ensure clear borders: the coral borders (microstructure) and pixel borders (nanostructures); ImageJ was used to measure their dimensions.

The corrosion behavior in open-to-air 3.5 wt.% NaCl solution was evaluated by potentiodynamic polarization and electrochemical impedance spectroscopy (EIS) tests performed at room temperature by using a BioLogic SP-300 potentiostat. A standard three electrode cell was used. The sample with surface area of 1 cm^2^ was used as the working electrode, a saturated Ag/AgCl electrode as the reference electrode and a platinum wire as the counter electrode. The potentiodynamic test was recorded with a scanning rate of 0.2 mV/s starting from the open circuit potential (OCP). The EIS test was performed at OCP with a voltage amplitude of 10 mV and a frequency range from 0.5 Hz to 10^5^ Hz. For all measurements, the working electrode was put in contact with the electrolyte solution until a steady state was reached (about 5–10 min). Three replicas were performed for each condition.

## 3. Results and Discussion

### 3.1. Morphologies

**Figure 1:**
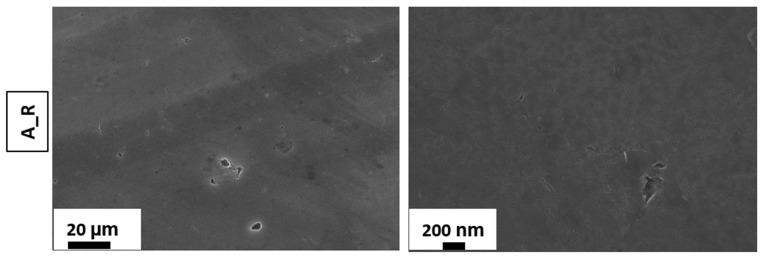
SEM images of the as-received sample.

**Figure 2:**
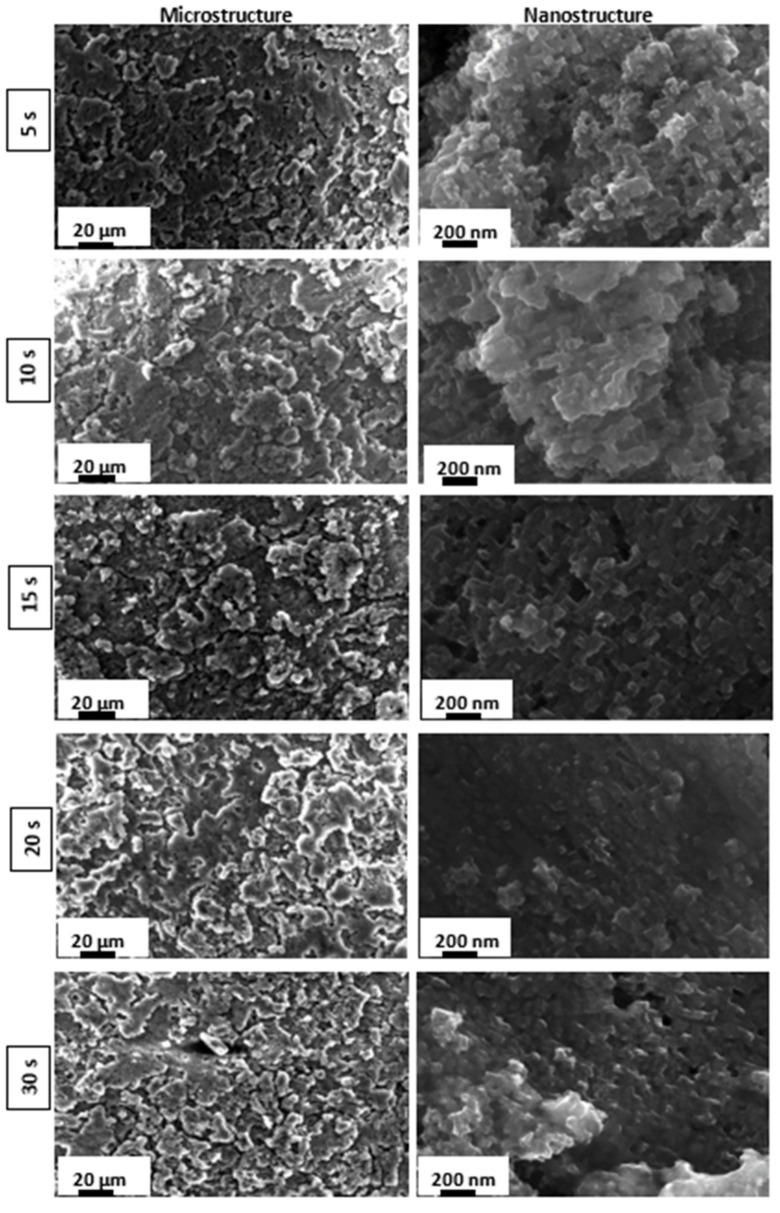
SEM images of superhydrophobic surfaces prepared by etching at different times.

Figure 1 and Figure 2 illustrate, respectively, the SEM images of the as-received and the etched AA 6082 surfaces at different times. According to [48,49], it can be observed that the etching time influences the surface morphology of the as-received aluminum substrates, promoting the transformation from a flat toward a micro/nano rough surface. 

Three different stages can be identified:

1. Short etching time: In the early stages of the etching process, within 5–10 s, micrometer-sized rough structures, comparable to a coral network, were formed. Furthermore, at nanometer-scale an irregular and porous morphology can be observed;
2. Intermediate etching time: At increasing etching times (15–20 s), the coral network microstructure is still preserved. The surface nanostructure becomes gradually regular, homogeneous and less porous, acquiring a morphology resembling a pixel-like structure;
3. Long etching time: Following a long etching time (30 s), the aluminum surface at nano-size level acquires a less evident but more irregular structure.

The etching procedure mostly involves an attack at the defect sites on the aluminum surface. The etchant can favor metal dissolution starting from defects and β’’ Mg_2_Si precipitates interface along crystallographic <100> directions, thus promoting a tunnelling corrosion morphology [50]. At low immersion times, the etching generates large micrometric steps. Then, dissolution proceeds through the tunnelling pathway inducing the formation of an irregular nanometric structure. This nanostructure continuously changes, becoming more regular when a slight increase in etching time is used. At a long etching time (30 s), a reduction in nano-patterning is observed, while remaining a mainly rough surface. 

The obtained nano/micro structure is due to the coupled action of the hydrofluoric and hydrochloric acids. In such a context, the use of this HF/HCl acidic solution (Beck’s solution) is a strongly recommended option in order to create a coupled uniform micro/nano rough structure [33,35,51]. Indeed, the presence of F-anions promotes the formation of a pseudo-passive film, probably a complex oxyfluoride film [52], in competition with the depassivating action of Cl^−^ anions [53]. However, when using only HCl acid, the aluminum surface cannot be uniformly and homogeneously etched. As shown by Lee et al. [48], the etching is effective only on some micrometric local portions (dimensions higher than 1 µm) requiring a long time for a complete etching of the surface. 

On the other hand, HF promotes the initial etching of the Si-rich impurities. When etching high purity aluminum (99.999%) by Beck’s solution, a non-uniform attack of the surface was obtained, while by etching an Al alloy surface a more homogeneous surface was achieved [48]. Furthermore, the etching action on the aluminum alloy using only HF starts after relatively high immersion times (30–50 min). However, adding a small quantity of this acid to other acids promotes the electrochemical dissolution of the alloy [54].

Therefore, a synergistic action of HF and HCl acids plays a relevant role in the effective micro- and nano-level etching of the aluminum alloy surface. Thus, the combination of the hydrofluoric and hydrochloric acid actions can be identified as a successful tool to create a hierarchical micro and nanostructure on an AA6082 surface.

**Figure 3:**
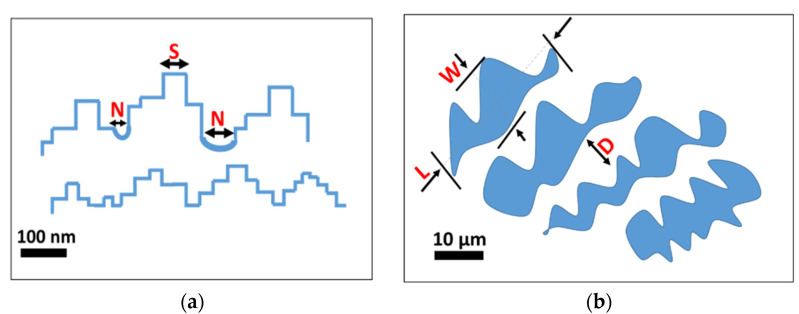
Schematic representation of the super-hydrophobic surface structures (**a**) geometrical parameters for nano-scale morphological characterization; (**b**) geometrical parameters for micro-scale morphological characterization.

**Figure 4:**
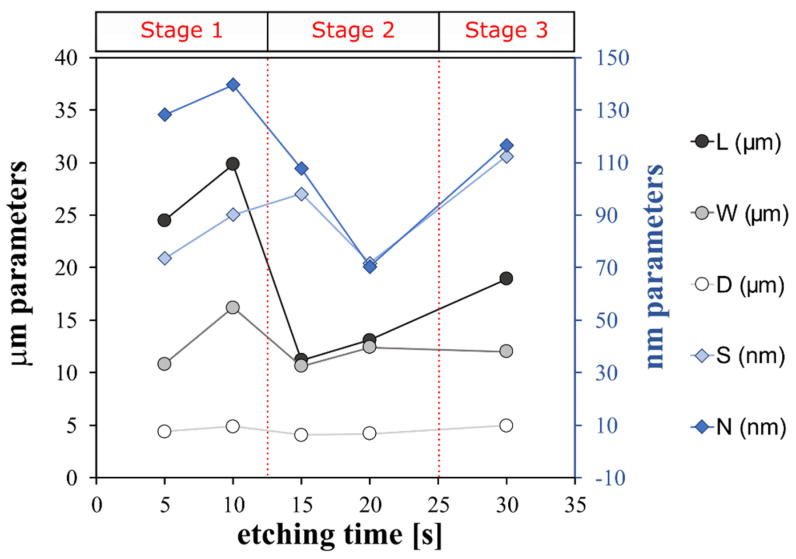
Structure features of superhydrophobic aluminum surfaces etched for different times.

An indirect qualitative analysis of the rough microstructure features of the treated surfaces was performed by digital image analysis of the SEM micrography. The contours of the coral colonies and the pixel cubes were manually selected and analyzed by using an image manipulation program (Gimp 2.10.12). Based on the SEM images, some geometrical parameters were identified as reference indices of the micro- and nano-scale morphologies. In particular, as shown in Figure 3, L and W are the length and the width of the coral colony, respectively. D is the distance between these colonies. L, W and D are identified as geometrical parameters characterizing the micro-scale morphology. S is the average of the pixel square sides and N is the neighbor distance. S and N are identified as geometrical parameters characterizing the nano-scale morphology. Details of the determined geometrical parameters for all etched surfaces are summarized in Figure 4. 

The trend of the geometric parameters over the increasing etching time indicates that the surface morphological structure progressively evolves, modifying its micro- and nano-texture distribution.

At micro-scale level, only A_FS15 and A_FS20 have L values that are similar to W ones and the lowest distances, D, between colonies (5–6 µm). This indicates an almost regular and homogenous surface micro-structure. At nano-scale level, the A_FS20 sample is also characterized by the lowest values for the S and N indices (~70 nm) indicating that this sample is characterized by the finest nanostructure. However, at the low etching time (A_FS5 and A_FS10) and long etching time (A_FS30) a mismatch on the morphological parameters can be highlighted suggesting an incomplete or over-etched surface texture, respectively. 

### 3.2. Wettability

To investigate the effect of the textured morphology on the wettability behavior of the aluminum alloy substrates, measurements of contact angles and sliding angles for the different preparation steps were performed [55,56,57]. 

**Figure 5:**
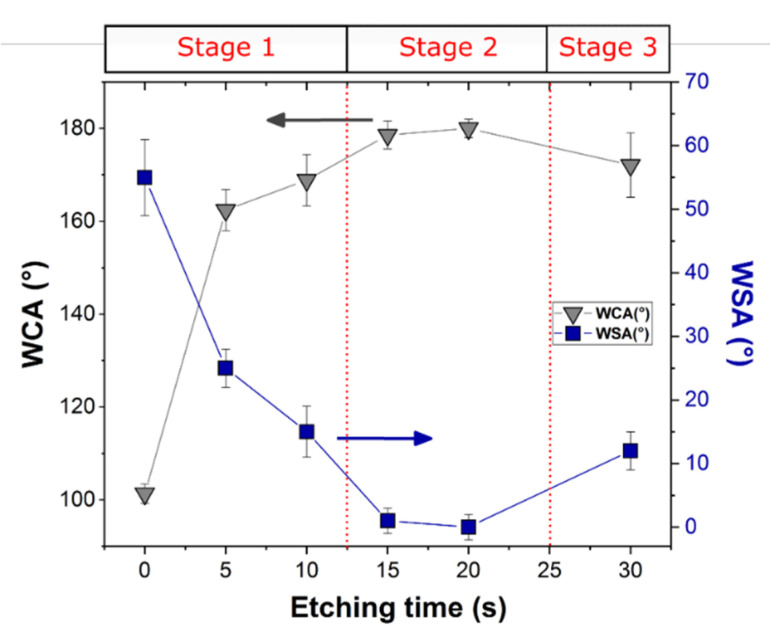
Water contact angles and sliding angles of aluminum alloy surfaces etched at different times after silanization.

Figure 5 illustrates the effect of etching time on the water contact angles (WCA) and the water sliding angles (WSA) of the silanized aluminum surfaces. 

Due to silane self-assembling on the surface, a clear increase in the WCA was observed, from around 69° (value not shown in Figure 5) to 101° from the A_R to A_RS samples, respectively. The hydrophobic organic alkyl chains anchored on the aluminum alloy surface positively affect the wetting behavior by greatly reducing its interaction with water [11,58,59,60], thus allowing the transition from a hydrophilic to a hydrophobic behavior. The coupled action of roughening and silanization effects can be evidenced on the other samples. Indeed, it is evident that the WCA increases with increasing etching time. The maximum WCA was exhibited for the surface etched for 20 s (~180°) (Figure S1 (Supplementary Materials)). After an etching time of 20 s, a slight reduction can be identified. To summarize, the WCA rises by about 11%, from 162.4° to around 180°, and the WSA decreases by 25% when the etching time increases from 5 s to 20 s. Finally, when the etching time reaches 30 s, the WCA diminishes by 10% and the WSA increases by around 4.4%, compared to that for 20 s. Therefore, these results indicate that the optimum super-hydrophobicity (high WCA and low WSA) was obtained at 20 s of etching time (A_FS20 batch).

All the etched samples can be roughly classified as superhydrophobic (WCA > 150°), although the WSA ranges from 25° to 0°, with the minimum for the A_FS20 batch. The observed changes in wettability behavior at increasing etching time can be attributed to the different interaction of the roughened silanized surface with the water. It is known that there are two types of superhydrophobic state of the surfaces: Cassie–Baxter and Wenzel states [56]. In the former, the dispersed protrusions of the surface profile can trap air in the cavities and thus the water droplet rests on the air layer and easily rolls off. The Cassie–Baxter surface is demonstrated in nature by Lotus leaves. On the other hand, in the Wenzel state, the water droplet penetrates the surface cavities resulting in high water adhesion (WSA > 90°). The typical representative of this state, in nature, is the rose petal. 

**Figure 6:**
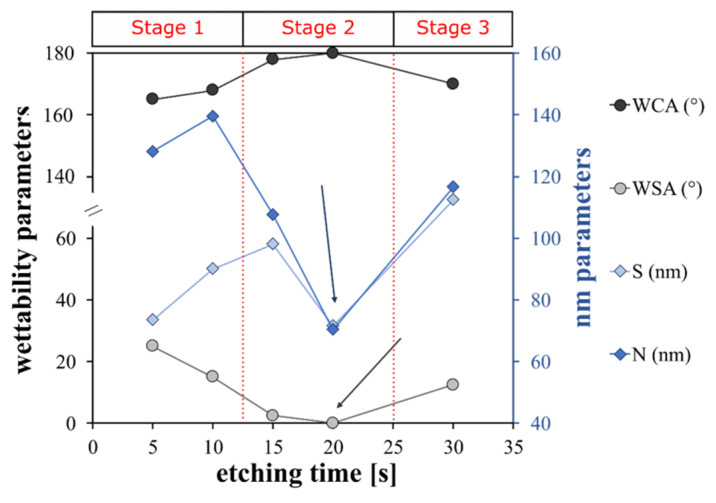
Correlation between wettability and surface parameters.

Therefore, the fraction of air trapped in the liquid/solid interface is a very important factor that controls the WCA and WSA and thus the superhydrophobic surface type. The results indicate that a transition between the Wenzel state and Cassie–Baxter state can occur when the etching time changes. The A_FS5 and A_FS10 samples are in an intermediate state between Wenzel and Cassie–Baxter as confirmed by the quite high sliding angles (25° and 15°, respectively), which indicates that the air is only partially entrapped into the surface cavities. However, A_FS15 and A_FS20 are characterized by Cassie–Baxter surfaces as indirectly identifiable by the low WSA (0 ≤ SA ≤ 10°). On the other hand, increasing the etching time to 30 s, the surface returns to the mixed Wenzel/Cassie–Baxter state (WSA = 12°), see Figure 6. 

In particular, the A_FS20 sample is characterized by the highest WCA (180°) and the lowest WSA (0°), which is probably due to the regular and homogeneous nano-protrusions formed on the micro-roughened profile that enhance the formation of a continuous air film between the metal–water interface, following the Cassie–Baxter state (Figure 6) [61]. Li et al. [62] confirmed that the achievement of an equilibrium between the large WCA and the small WSA requires a dual scale structure with a thin solid fraction. When the etching duration is lengthened to 30 s, the ordered nanostructure is destroyed, as shown by the SEM analysis (Figure 2) which may negatively affect the air layer. In fact, the air fraction trapped on the solid surface of the A_FS30 is lower than the A_FS20 sample resulting in a lower WCA and higher WSA. Thus, at 30 s, the inverse transition from Cassie–Baxter to Wenzel can occur [63].

### 3.3. Anti-Corrosion Behavior

The study of the corrosion behavior of these surfaces was undertaken to assess their industrial applicability in the marine field. Surface hydrophobicity significantly affects the interaction of the metal surface with water, thus affecting its electrochemical behavior.

**Figure 7:**
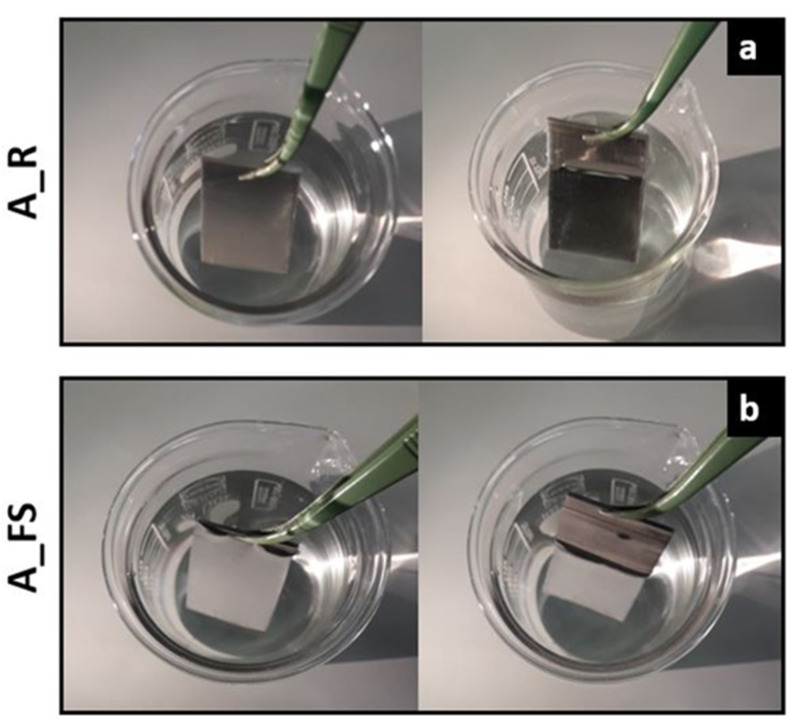
Photographs of as-received Al alloy samples (A_R) (**a**) and superhydrophobic samples (A_FS20) (**b**) immersed in a 3.5 wt.% NaCl aqueous solution.

For a qualitative evaluation of the water repellency of the textured silanized surfaces compared to the as-received one, Figure 7 shows some pictures of as-received aluminum (A_R) (a) and superhydrophobic (A_FS20) (b) samples immersed in 3.5 wt.% NaCl solution.

Notably, when the superhydrophobic surface is immersed in the solution, the surface is remarkably bright when viewed from an oblique angle (Figure 7b) suggesting the presence of an air cushion entrapped on the rough superhydrophobic surface according to the theory of total reflection [63,64]. In fact, air is optically thinner and less dense than water. When light travels from the water to the air interface with an incidence angle higher than the critical 49°, it can be totally reflected. The presence of trapped air can influence the interaction of the metal with the electrolyte solution, representing a key factor in the anticorrosion capabilities of the surface. 

The ability of the superhydrophobic surfaces to protect the 6082 aluminum alloy from corrosion in simulated seawater (3.5 wt.% NaCl solution) was evaluated by electrochemical tests. In particular, the potentiodynamic test could, preliminarily, give a comparative assessment of the electrochemical behavior of all the batches. 

**Figure 8:**
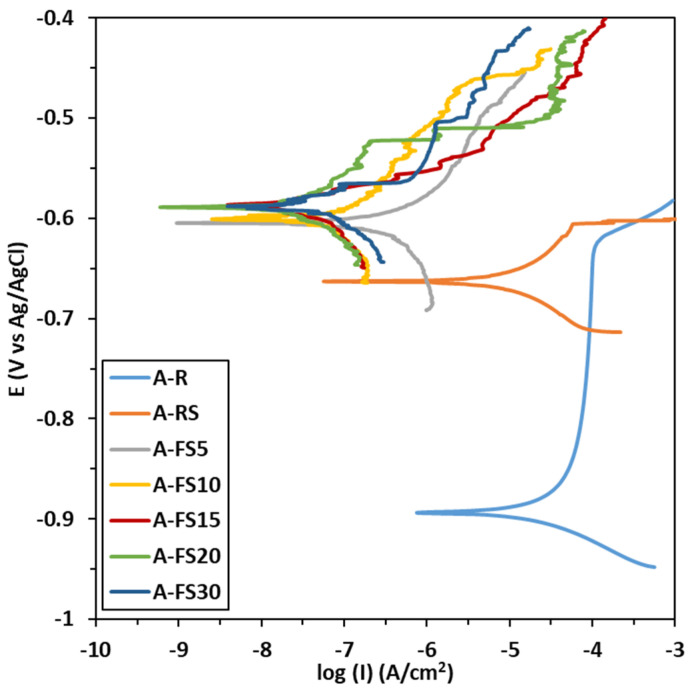
Polarization curves of AA6082 samples immersed in 3.5 wt.% NaCl aqueous solution.

The potentiodynamic polarization curves of the aluminum alloys samples treated at different times (A_FS5, A_FS10, A_FS15, A_FS20, and A_FS30), compared to the as received aluminum substrate (A_R) and the chemically modified aluminum substrate with silane (A_RS) are shown in Figure 8. The corrosion current density (i_cor_) and the corrosion potential (E_corr_) derived from the potentiodynamic polarization curves using the Tafel extrapolation method are summarized in Table 3. 

Generally, a surface with high corrosion potential and low corrosion current has superior corrosion resistance [65]. It can be seen that the corrosion potential of the aluminum alloy positively increases from around −904 mV to about −600 mV thanks to the superhydrophobic behavior. All the etched surfaces evidenced a higher OCP than the A_R and A_RS samples.

Analogously, further consideration can be given to evaluating the corrosion current evolution at varying surface hydrophilization levels. The untreated aluminum alloy is characterized by low corrosion resistance (i_cor_ = 2.2 × 10^−5^ A/cm^2^) and low breakdown potential observed at about −620 mV vs Ag/AgCl_sat_. However, due to the etching treatment, the corrosion current density decreases by about four orders of magnitude. The etched silanized samples show a corrosion current ranging from 3.3 × 10^−7^ A/cm^2^ for A_FS5 to 7.1 × 10^−8^ A/cm^2^ for A_FS20. The values of the corrosion current densities of the superhydrophobic samples are less than those reported in the earlier literature [9,66].

These results indicate that the corrosion rate of the aluminum alloy decreases when increasing the surface hydrophobicity. The values of the WSA are consistent with the corrosion current trend, highlighting a higher corrosion resistance for the A_FS15, A_FS20 and A_FS30 batches characterized by the lowest WSA (preserving the A_FS20 batch as the best option). At the same time a clear increase of about 100 mV in breakdown potential can be identified by comparing the polarization curves of the textured silanized surfaces and the as-received one.

On a superhydrophobic surface, the air can be trapped into the hierarchical rough structure and acts as a cushion between this surface and the simulated seawater. This air cushion prevents the diffusion of the aggressive electrolyte toward the metal substrate. The coupled effect of the trapped air and the self-assembled silane layer can form a double-layer protective system thus enhancing surface corrosion resistance. Conversely, for the as-received sample, the corrosive ions are probably able to rapidly interact with the surface and easily penetrate it, resulting in lower corrosion resistance. Thus, the combined action of surface texturing and silanization plays a decisive role in obtaining an excellent corrosion resistance.

**Figure 9:**
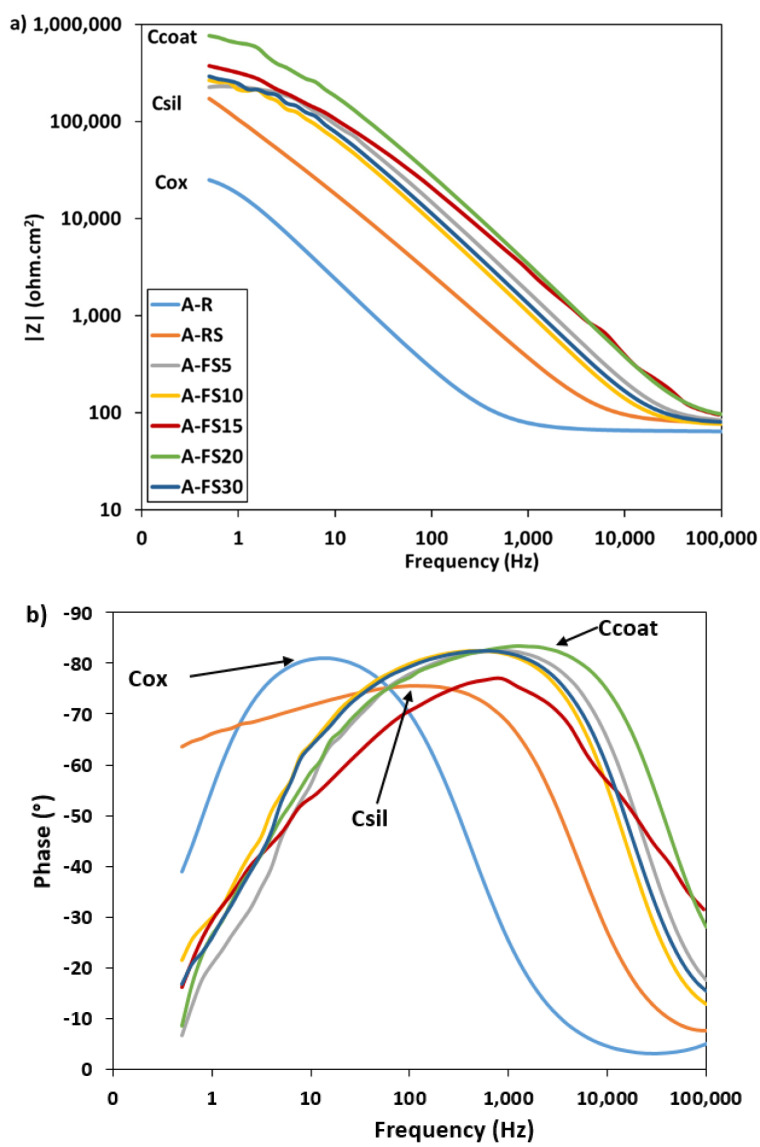
(**a**) Bode modulus and (**b**) phase plots of the as-received aluminum alloy and the superhydrophobic samples.

In addition, impedance spectroscopy analysis was performed to provide further information on the surface properties. Figure 9 shows the evolution of the impedance spectra for increasing etching times. For comparison purposes, the A_R and the A_RS are also reported. At low frequencies, all curves exhibit a small plateau (Figure 9a). The plateau in the as-received sample spectrum is ascribed to the thin aluminum oxide surface, R_ox_ (~2.5 × 10^4^ Ω × cm^2^ at 0.5 Hz). The silanized sample (A_RS) clearly shows a mainly capacitive behavior (evidenced by a linear shift in the modulus trend on the right toward higher frequencies). At the same time, an evident increase in impedance magnitude in all the frequency ranges can be highlighted. After the creation of both the rough hierarchical nano/microstructure and the self-assembled silane coating on the aluminum surface, an increase in the |Z| of about one order of magnitude is observed. All curves show a less evident stabilization of |Z| at low frequency, ascribed to the effect of the self-assembled silane layer deposition and entrapped air cushion, R_coat_ [67]. The highest magnitude of impedance modulus, about ~7.7 × 10^5^ Ω × cm^2^ at low frequency range, was observed for the A_FS20 batch. By evaluating the phase plot in Figure 9b, it is possible to identify an apparent single time constant for all batches. The as-received sample, A_R, exhibits a peak in the phase angle at low frequency (~10^1^ Hz) that can be associated to the aluminum oxide layer (C_ox_) [68]. 

The EIS spectra of the A_FS samples are characterized by a wide phase peak at a high frequency. A time constant can be identified at high frequency (~10^3^ Hz), in correspondence with the phase peak. This trend is typical of the capacitive behavior of external protective layers [69]. In addition, the phase angle near to −90° indicates that this protective film provides a good barrier action. This behavior can be ascribed to the coupled action of the surface texturing and silanization. The surface texturing shifts the peak at very high frequency, probably related to the formation of a protective layer of air according to the Cassie–Baxter regime (C_coat_) and the barrier capacity of the silane layer (C_sil_). Furthermore, the presence of a secondary small peak at about 10 Hz, ascribed to the oxide layer, can be identified.

As mentioned above, for increasing the etching times, the nanoscale pixel-like structure becomes gradually regular and homogeneous which improved the chances for air to be trapped in the hierarchical rough surface forming a fairly continuous layer. This air layer can work as the dielectric for a parallel plate capacitor, that prevents the electrons from moving between the electrolyte and the aluminum surface and thus enhances corrosion inhibition [70]. These results are consistent with wettability measurements, where the CA obtained on the aluminum surfaces follows the order A_FS20 > A_FS15 > A_FS30 > A_FS10 > A_FS5. This confirms that the increase in the water contact angle (increase in hydrophobicity) leads to the enhancement of the corrosion resistance. The etching time acts synergistically on the super-hydrophobicity and corrosion resistance behavior. Thus, these results clarify a relationship among micro-nano hierarchical morphology, water contact/sliding angles and corrosion resistance, providing a new vision for an improvement of knowledge on the choice of a suitable surface finish for the preparation of corrosion-resistant surfaces coupling micro-nano textured superhydrophobic surfaces.

## 4. Conclusions

Aluminum superhydrophobic surfaces were fabricated using an economic and simple process. These surfaces exhibited different morphologies and wettability because of the variation in the etching time in Beck’s solution. Several morphological parameters were utilized to explore the correlations between the microstructural features and the wetting behavior. The key results are as follows:

- The mechanism underlying the wetting alteration is based on the distribution of the peaks and valleys on the aluminum surface;
- The wetting transitions from the Cassie–Baxter to the Wenzel regime and from Wenzel to Cassie–Baxter can be controlled by changing the etching time;
- A relationship between water contact/sliding angles and surface morphology was evidenced, offering new insight into the fabrication of superhydrophobic surfaces with controlled levels of performance;
- The sample etched for 20 s is characterized by the greatest water repellency behavior (WCA = 180°; WSA = 0°) and the best corrosion inhibition (|Z| more than two orders of magnitude higher than for the untreated aluminum).

Finally, the long-term durability and the modeling of the corrosion behavior of the developed superhydrophobic surfaces in severe environmental conditions will be explored as the scope of future research works.

## Figures and Tables

**Table 1:** Element composition of the alloy AW-6082 T6 obtained by EDS analysis.

Chemical Element	Content (wt.%)
Silicon (Si)	1.06
Magnesium (Mg)	1.35
Manganese (Mn)	0.26
Iron (Fe)	0.3
Aluminum (Al)	Balance

**Table 2:** Samples codes and preparation details.

Code	Step 1: Etching	Step 2: Silanization
A_R	–	-
A_RS	–	S18
A_F5	5 s	-
A_FS5	5 s	S18
A_F10	10 s	-
A_FS10	10 s	S18
A_F15	15 s	-
A_FS15	15 s	S18
A_F20	20 s	-
A_FS20	20 s	S18
A_F30	30 s	-
A_FS30	30 s	S18

**Table 3:** Electrochemical parameters for the corrosion of different samples immersed in 3.5 wt.% NaCl aqueous solution.

Samples	A_R	A_RS	A_FS5	A_FS10	A_FS15	A_FS20	A_FS30
E_corr_ (mV)	−904	−661	−609	−603	−578	−583	−592
I_cor_ (i/A·cm^−2^)	2.2 × 10^−5^	7.9 × 10^−6^	3.3 × 10^−7^	1.5 × 10^−7^	8.5 × 10^−8^	7.1 × 10^−8^	7.7 × 10^−8^
E_break_ (mV)	−620	−610	−498	−484	−560	−516	−568

## Data Availability

The data presented in this study are available on request from the corresponding author.

## Supplementary Materials

The following supporting information can be downloaded at: https://www.mdpi.com/article/10.3390/ma15238549/s1, Figure S1: (1)–(6) corresponding to the sequence of snapshots of droplets (5 µL) in the surface of A_FS20.
