# Supplementary material for: Anticorrosion Superhydrophobic Surfaces on AA6082 Aluminum Alloy by HF/HCl Texturing and Self-Assembling of Silane Monolayer
Source: Materials (Basel). 2022 Nov 30;15(23):8549. doi: 10.3390/ma15238549 (PMC9740352; doi:10.3390/ma15238549)
Supplement: Supplementary file 1 [file materials-15-08549-s001.zip › materials-1956217-supplementary.pdf]

materials

Supplementary Material

Anticorrosion Superhydrophobic Surfaces on AA6082 Aluminum Alloy by HF/HCl Texturing and Self-Assembling of Silane Monolayer

Amani Khaskhoussi *, Luigi Calabrese * and Edoardo Proverbio

Department of Engineering, University of Messina, Contrada di Dio Sant'Agata, 98166 Messina, Italy
* Correspondence: amani.khaskhoussi@unime.it (A.K.); lcalabrese@unime.it (L.C.)

On the surface of A_FS20 sample, the water droplets roll off immediately without any adhesion and without tilting, showing excellent water repellency (WCA = 180°, WSA = 0). The Figure S1 shows time-steps of the free-falling process of the water droplet on the surface of the sample having WCA = 180° and WSA = 0°. The initial state (1) revealed the distance between the water droplet and the Al treated substrate. In fact, the droplet shows a natural appearance due to the self-gravity. After 2 ms, the water droplet fell down (2) and hit the substrate at the speed of 500 mm·s^{-1} after 4 ms (3). Subsequently, the water droplet fully rebounded upward (4) and elastically bounced one time before rolling off the Al treated substrate without any residual water.

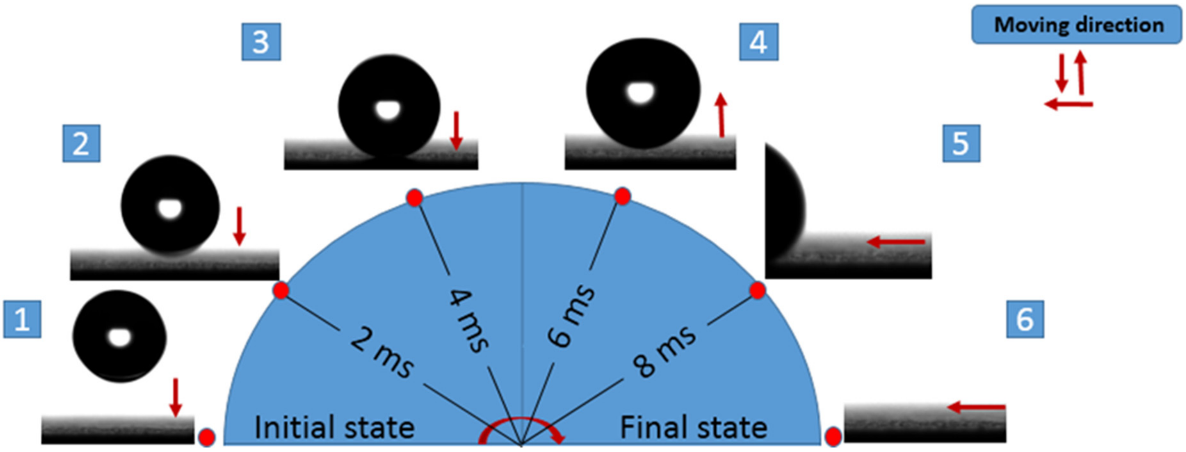

Figure S1. (1)–(6) corresponding to the sequence of snapshots of droplets (5 µL) in the surface of A_FS20.
